# Bridging Ethnobotanical Knowledge and Multi-Omics Approaches for Plant-Derived Natural Product Discovery

**DOI:** 10.3390/metabo15060362

**Published:** 2025-05-29

**Authors:** Kekeletso H. Chele, Lizelle A. Piater, Justin J. J. van der Hooft, Fidele Tugizimana

**Affiliations:** 1 Department of Biochemistry, University of Johannesburg, Auckland Park, Johannesburg 2006, South Africa; 215024679@student.uj.ac.za (K.H.C.); lpiater@uj.ac.za (L.A.P.); 2 Bioinformatics Group, Wageningen University & Research, 6708 PB Wageningen, The Netherlands; 3 International R&D, Omnia Group, Johannesburg 2191, South Africa

**Keywords:** computational tools, ethnobotany, metabolite annotation, metabolite identification, metabologenomics, metabolomics, natural products, paired omics, traditional medicine

## Abstract

For centuries, plant-derived natural products (NPs) have been fundamental to traditional medicine, providing essential therapeutic compounds. Ethnobotanical knowledge has historically guided NP discovery, leading to the identification of key pharmaceuticals such as aspirin, morphine, and artemisinin. However, conventional bioactivity-guided fractionation methods for NP isolation are labour-intensive and can result in the loss of bioactive properties due to the focus on a single compound. Advances in omics sciences—genomics, transcriptomics, proteomics, metabolomics, and phenomics—coupled with computational tools have altogether revolutionised NP research by enabling high-throughput screening and more precise compound identification. This review explores how integrating traditional medicinal knowledge with multi-omics strategies enhances NP discovery. We highlight emerging bioinformatics tools, mass spectrometry techniques, and metabologenomics approaches that accelerate the identification, annotation, and functional characterisation of plant-derived metabolites. Additionally, we discuss challenges in omics data integration and propose strategies to harness ethnobotanical knowledge for targeted NP discovery and drug development. By combining traditional wisdom with modern scientific advancements, this integrated approach paves the way for novel therapeutic discoveries and the sustainable utilisation of medicinal plants.

## 1. Introduction

The study of natural products (NPs) has its roots in the 19th century, when organic chemistry principles were first established. NPs encompass a diverse range of bioactive compounds derived from plants, bacteria, fungi, and animals, categorised into primary metabolites (essential for survival) and secondary metabolites (specialised compounds aiding adaptation and defence) [1,2]. Over millennia of evolution, secondary metabolites have played crucial roles in biotic and abiotic interactions, functioning as antioxidants, antibiotics, and enzyme modulators [3]. These include phenolic acids, flavonoids, alkaloids, and steroids, many of which have significant pharmaceutical applications [4,5]. In the context of human health, the immense coevolution abilities of NPs have driven their extensive use in medicine, from pain relief to disease treatment [6,7]. Ethnobotanical knowledge has led to the discovery of key pharmaceuticals such as aspirin (*Salix* spp.), morphine (*Papaver somniferum*), quinine (*Cinchona* spp.), and artemisinin (*Artemisia annua*) [8]. Today, this knowledge continues to inspire modern drug discovery, contributing significantly to the global pharmaceutical industry. The annual medicine market is valued at approximately 1.1 trillion US dollars, with 35–50% of all approved drugs derived from natural sources, including plants (25%), microorganisms (13%), and marine organisms [9]. From 1981 to 2019, the United States of America (USA) Food and Drug Administration (FDA) has approved numerous NP-based drugs [10], such as cyclosporin (immune suppressant) [11], bleomycin (cancer therapy) [12], and galantamine (Alzheimer’s treatment) [13].

Despite their immense potential, NP research faces challenges, including (i) limited understanding of biosynthetic pathways, which impedes discovery and engineering of new NPs [14], (ii) the lack of standardised and reproducible isolation methods, leading to difficulties in comparing and reproducing results, thereby hampering collaboration [15], (iii) limited knowledge about structure–activity relations (SAR) due to the complexity and diverse chemical properties of NPs, (iv) the challenge of cultivating many microorganisms in the laboratory environment [16], (v) mining the silent biosynthetic gene clusters which could potentially code for NPs under laboratory conditions [17], and (vi) difficulties in structural elucidation of NPs, which often require advanced analytical techniques such as nuclear magnetic resonance (NMR) and mass spectrometry (MS) to decode complex molecular structures accurately. These challenges stress the importance of a holistic approach that blends traditional expertise with modern scientific advancements.

The traditional drug discovery process has been slow, producing only a limited number of new clinically approved molecules per decade [18]. Given the urgent global demand for novel therapeutics, particularly against emerging and neglected diseases, plants offer a vast reservoir of pharmacologically active compounds. Unlike synthetic molecules, plant-derived NPs have evolved for optimal biological interactions, making them prime candidates for drug development [6,19]. Their biosynthetic complexity often surpasses what can be achieved synthetically, reinforcing the value of ethnobotany in modern pharmaceutical exploration. This review delves into the evolution of plant-derived NPs, from their ethnobotanical roots to modern scientific applications. It explores the role of computational metabolomics, multi-omics integration (genomics, transcriptomics, proteomics, and metabolomics), and computational tools in overcoming key NP research challenges. By bridging traditional knowledge with cutting-edge science, NP research holds the potential to revolutionise global health through next-generation drug discovery.

## 2. Plant-Derived Natural Products

### 2.1. Traditional Medicine Based on Plant-Derived Natural Products

“In a world driven by technology and innovation, we must not overlook the wisdom of our ancestors”—Anonymous. As this statement underlines, since prehistoric times, natural compounds from plants have been the backbone of traditional healing across cultures and have shaped history and culture [20]. Fossil records reveal that plant-based medicinal practices date back at least 60,000 years, representing an evolving repository of effective treatments and limitations [21]. Such traditional medicines stand as the oldest form of healthcare, with plant-based treatments addressing both mental and physical illnesses. The examples in Table 1 highlight plants chosen for their historical significance and wide use in traditional medicine systems, including well-documented treatments that have since been incorporated into modern medicine. These traditional systems, such as traditional Chinese medicine (TCM), Ayurveda, and African healing practices, offer a rich knowledge base that has guided the discovery of numerous bioactive molecules still in use today.

Historically, healers and practitioners identified medicinal plants based on empirical knowledge, using trial and error, intuition, spiritual beliefs, and sensory cues like taste, smell, and colour [20,22]. These classifications also considered the energetic qualities of plants, such as their warming or cooling effects on the body. Over time, this knowledge was codified in foundational texts like the Charaka Samhita in Ayurveda and the Compendium of Materia Medica in TCM [23,24]. The Charaka Samhita, dating back to the second century before common era/before Christ (BCE/BC), provides detailed guidance on internal medicine, emphasising the balance of the body’s three doshas for maintaining health [25]. Similarly, the Compendium of Materia Medica, compiled in the 16th century CE by Li Shizhen, is a comprehensive work on Chinese herbal medicine, cataloguing over 1800 medicinal substances and detailing their properties, uses, and preparations [24,26]. Both texts remain relevant in their respective medical traditions and continue to influence modern herbal medicine practices. As societies evolved, many of these plant-based remedies have been validated through scientific research and now contribute to modern pharmacology.

As highlighted in Table 1, traditional medicine practices vary significantly across different regions, including Asia, Africa, and America, with each region using specific plants and preparation methods tailored to their unique cultural contexts. While traditional medicine in some countries, such as Australia, faces challenges like the erosion of traditional knowledge, cultural shifts, and limited healthcare access, 80% of the population in Africa continues to rely on traditional medicine, either as a primary treatment or alongside modern medical practices [20,27]. In Africa, traditional healers (*sangomas* or *inyanga*) continue to use plants such as *Sutherlandia frutescens* (cancer bush) for immune stimulation and chronic disease management [28]. Similarly, *Artemisia afra* (African wormwood) has long been used for its antimalarial, antimicrobial, and anti-inflammatory properties [29]. These practices, which have survived centuries of change, highlight the continued relevance of traditional medicine in many societies today. Table 1 showcases several other examples of plants with well-documented medicinal use across various cultures. Notable cases include *Papaver somniferum* (opium poppy), which has been used for pain relief since ancient times, leading to the isolation of morphine, the first bioactive natural product [30]. *Artemisia annua* (Sweet wormwood), used in traditional Chinese medicine to treat fever, was the source of the discovery of artemisinin, a powerful antimalarial agent [31]. These examples illustrate how traditional medicine has directly influenced modern drug discovery by providing the basis for bioactive molecule identification and clinical applications.

**Table 1:** Selection of well-documented plants used traditionally for medicinal purposes in African, Chinese, American, and Indian communities.

Plant Name	Tissue Used	Administration	Medicinal Use/Treatment	Reference
Africa				
***Acacia senegal*** **(gum arabic)**	Whole plant	Oral, topical	Bleeding, bronchitis, diarrhoea, gonorrhoea, leprosy, typhoid fever, upper respiratory tract infections	[32]
***Aloe ferox*** **(bitter aloe or Cape aloe)**	Leaves	Oral	Anti-inflammatory, analgesic, calming, antiseptic, germicidal, antiviral, antiparasitic, anticancer	[33]
***Artemisia herba-alba*** **(wormwood)**	Leaves, stems	Oral	Arterial hypertension, diabetes, bronchitis, diarrhoea, hypertension, neuralgias	[34]
***Catharanthus roseus*** **(Madagascar periwinkle)**	Leaves, seeds, stems, petals	Oral	Anticancer, rheumatism, skin disorders, venereal diseases	[35]
China				
***Aconitum napellus*** **(monkshood)**	Roots	Oral, topical, inhalation	Hypertension, haemorrhoids, colic, upper urinary tract cancer, kidney failure	[36]
***Trichosanthes kirilowii*** **(Chinese cucumber)**	Seeds, fruits, pericarps, roots	Oral	Tumours, reduces fevers, swelling and coughing, abscesses, amenorrhea, jaundice, polyuria	[37]
***Chrysanthemum* spp.** **(mums)**	Flowers, seeds	Oral, topical	Chest pain, high blood pressure, type 2 diabetes, fever, cold, headache, dizziness, and swelling	[38]
***Panax ginseng*** **(ginseng)**	Leaves, stems, root	Oral	Fatigue, stress, asthma, cancer, diarrhoea, anxiety, mental health	[39]
***Artemisia annua*** **(sweet wormwood)**	Leaves	Oral	Malaria, fever reduction, inflammation	[40]
America				
***Allium sativum*** **(garlic)**	Cloves	Oral	Hypercholesterolemia, claudication, common cold, osteoarthritis	[41]
***Hypericum perforatum*** **(St. John’s wort)**	Leaves, flowers, seeds	Oral	Depression, menopausal symptoms, attention-deficit hyperactivity disorder (ADHD)	[42]
***Berberis vulgarisn*** **(barberry)**	Fruit, bark, root, stem	Oral	Fever, cough, liver disease, depression, hyperlipidaemia, hyperglycaemia and bleeding	[43]
***Echinacea purpurea*** **(purple coneflower)**	Leaves, flower petals	Topical, oral	Infections, wounds, urinary tract infections, cold and flu	[44]
***Taxus brevifolia*** **(Pacific yew)**	Bark	Oral	Breast and ovarian cancer	[45]
***Digitalis lanata*** **(woolly foxglove)**	Leaves	Oral	Heart failure, arrhythmia, atrial fibrillation	[46]
India				
***Terminalia arjuna*** **(Arjuna)**	Stem, bark, fruits leaves	Oral	Fractures, ulcers, antibacterial, antimicrobial, antioxidant, antiallergic, antifertility, anti-HIV	[47]
***Andrographis paniculate*** **(Kalmegh)**	Whole plant	Oral	Cold, diarrhoea, fever, jaundice, as a health tonic for the liver and cardiovascular health, antioxidant	[48]
***Mucuna Pruriens*** **(Kauch)**	Fruits, seed	Oral	Parkinson disease, sexual disorders	[49]
***Acacia catechu*** **(Khair)**	Leaves, bark, wood	Oral	Mouth ulcer, anaemia, high blood pressure, dysentery, colitis, gastric problems, bronchial asthma, cough, leucorrhoea and leprosy	[50]
***Catharanthus roseus*** **(periwinkle)**	Whole plant	Oral	Cancer, diabetes, vaginal discharge, tonsillitis, chest pain, high blood pressure, sore throat, intestinal pain, inflammation, toothache	[51]
***Papaver somniferum*** **(opium poppy)**	Seeds	Oral, injection	Pain relief, cough suppressant, diarrhoea	[30]

Traditional medicine systems utilise a diverse array of plant materials, including roots, leaves, bark, flowers, seeds, and fruits, each selected for specific therapeutic properties (Table 1) [20,21]. These materials are prepared in various forms, such as infusions, decoctions, tinctures, powders, and oils, influenced by cultural practices and desired therapeutic effects. In Asia, traditional Chinese medicine (TCM) emphasises holistic healing, often combining multiple plant extracts to restore balance [52,53]. Ayurvedic medicine classifies plants based on doshas (body energies) and uses them in powders and pastes, applied topically or orally [54,55]. In Africa, medicinal plants like *Sutherlandia frutescens* (cancer bush) are prepared as teas or poultices for immune support and chronic disease management [22,56,57]. Traditional African medicine is deeply intertwined with spiritual beliefs, often practiced by *sangomas* or *inyangas* in community-centred rituals. In Europe, particularly in the Mediterranean, the use of herbs like *Thymus vulgaris* (thyme) and *Salvia officinalis* (sage) has been integrated into folk medicine, often for treatment of common ailments like digestive issues and respiratory conditions [58,59,60]. While preparation methods and philosophies differ, traditional medicine systems share a common reliance on plant-based therapies, reflecting a rich cultural and medicinal heritage passed down through generations.

Despite its extensive history and continued use, traditional medicine faces significant controversy, particularly due to the lack of scientific validation for many of its remedies. This raises concerns about safety, efficacy, and the potential risks associated with treatments that have not undergone rigorous empirical testing [61]. Critics emphasise the difficulty of assessing both the risks and benefits of these treatments without standardised research protocols. Furthermore, ethical concerns arise when traditional practices involve the use of endangered species, potentially conflicting with global conservation efforts [22,62]. Thus, the integration of traditional and modern medicine presents a multifaceted opportunity. On the one hand, traditional medicine offers accessible and culturally significant healthcare options, especially in regions where access to modern medical infrastructure is limited. On the other hand, modern medicine provides a framework for scientific validation, standardisation, and the scalability of treatments through advanced pharmacological methodologies. The complementary strengths of these systems lie in the empirical knowledge and deep-rooted historical practices of traditional medicine, paired with the rigor, reproducibility, and evidence-based approach of modern medicine. When harmonised, these systems can foster innovative healthcare solutions that benefit from both the wisdom of traditional practices and the precision of modern scientific research. Understanding the chemical characteristics of these NPs is crucial for unlocking their medicinal properties and optimising their use in drug development. The following section explores the chemical diversity and structural complexity of plant-derived NPs, which contribute to their unique pharmacological effects.

### 2.2. Chemical Characteristics of Plant-Derived Natural Products

“Plants rule the planet”—[63]. Plants make up the majority of the planet’s living biomass, with approximately 450,000 known species responsible for 80% (~450 gigatons of carbon, Gt C; 1 Gt C = 1015 g of carbon) of the total biomass across all taxa on the biosphere (~550 Gt C) [64,65]. Over the centuries, plant-derived NPs have been utilised externally in the form of drugs, flavours, fragrances, antioxidants, dyes, pheromones, as well as insecticides [66]. According to the 2022 Global Plant Extract Market Analyses, plant extracts used as commercial commodities in the nutraceutical, pharmaceutical, and cosmeceutical fields were approximated to be worth USD 25 billion in 2022, USD 28 billion in 2023, and are estimated to reach USD 39 billion by 2027 at a compound annual growth rate of 9.0%. Due to these economical and pharmaceutical benefits and predictions, the plant-derived NP research has gained traction in the past decades.

The COCONUT database (https://coconut.naturalproducts.net/; accessed on 10 November 2024) reports over 400,000 plant-derived NPs, including secondary metabolites and other bioactive compounds. Many of these NPs have led to relevant pharmaceutical discoveries. Notable examples include morphine, an analgesic derived from *Papaver somniferum*, first isolated by Friedrich Sertürner in the 18th century [67], and digitalis, extracted from *Digitalis purpurea* by William Withering, which remains a key cardiac treatment [68,69,70]. Similarly, artemisinin, a potent anti-malarial compound isolated from *Artemisia annua*, was discovered in the 19th century by Tu Youyou, inspired by traditional Chinese medicine (TCM) [71,72]. Beyond these well-known examples, South African medicinal plants have also yielded important NPs. *Sutherlandia frutescens* (cancer bush) contains canavanine and pinitol, compounds with anti-inflammatory and immune-boosting properties [28]. *Agathosma betulina* (Buchu) produces limonene and menthone, traditionally used for their diuretic and antiseptic effects [73]. Within plants, these metabolites serve essential roles, including protection against environmental stressors, internal signalling, and interspecies communication. Such secondary metabolic pathways enable plants to produce bioactive compounds with chemical diversity, reflecting their diverse ecological roles and evolutionary adaptations, many of which have been extensively researched and successfully adapted for human medicine [74]. These specialised metabolites are broadly classified based on their chemical structures and biosynthetic origins into major classes, including alkaloids, terpenoids, flavonoids, and phenolics. This diverse plant chemistry encompasses a wide range of pharmacologically active molecules, as outlined in Table 2.

**Table 2:** Plant-derived bioactive compounds with their respective plant sources and suggested mechanism of action selected for their therapeutic relevance and extensive research support, including clinical validation.

Plant Source	Bioactive Compound	Compound Class	Effects/Bioactivity	Mechanism of Action	Reference
***Artemisia glabella***	Arglabin	Terpene	Antitumor	Inhibits farnesyl transferase	[75]
***Cannabis sativa***	Cannabidiol	Cannabinoid	Anti-epileptic, anxiolytic, antipsychotic, and anticancer	Modulates CB1, CB2, 5HT1A receptors in the central nervous system	[76]
***Capsicum annum***	Capsaicin	Alkaloid	Chronic pain syndromes such as postherpetic neuralgia and musculoskeletal pain	Activates transient receptor potential vanilloid 1 (TRPV1) in sensory nerves	[77]
***Colchicum* spp.**	Colchicine	Alkaloid	Gout	Scavenges reactive oxygen and nitrogen species, inhibits NF-kB, modulates activities of glutathione, catalase, and superoxide dismutase	[78]
***Genista tinctoria***	Genistein	Flavonoid	Anticancer, Alzheimer’s disease	Inhibits protein-tyrosine kinase, induces apoptosis, antimetastatic and antiangiogenic activity, antioxidant	[79]
***Gossypium hirsutum***	Gossypol	Terpene	Anti-infertility/male contraceptive, anticancer, antiviral, antimicrobial, antioxidant activities	Bcl-2 and sperm production inhibition, induces apoptosis, inhibits DNA polymerase and topoisomerase II	[80]
***Tabebuia avellanedae***	β-Lapachone	Quinone	Variety of cancers, especially solid tumours, anti-trypanosoma, antimicrobial, and antimalarial activities	Anticancer activity through formation of ROS in NQO1-positive cells, inhibits topoisomerase, modulates the mTOR pathway	[81]
***Larrea tridentate***	Masoprocol	Phenolic compound	Antineoplastic agent used in cancer chemotherapy	Inhibits 5-Lipoxygenase	[6]
***Podophyllum emodi***	Podophyllotoxin	Phenolic compound	Antitumour	Suppresses formation of mitotic spindles microtubules, cycle arrest via polymerisation of tubulin	[82]

A comprehensive examination of plant-derived NPs, highlighting their diverse chemical structures and biological roles, provides a crucial foundation for understanding their vast potential across various applications. Having established the importance of these compounds in both natural ecosystems and human health, it is essential to reflect on the traditional methodologies used to study and harness these valuable resources. These classical approaches have paved the way for modern advancements in natural product research, offering the initial insights and frameworks that continue to shape contemporary scientific exploration and drug discovery. The isolation and purification of plant-derived natural products typically involve a combination of physical and chemical techniques. These include solvent extraction, solid-phase-extraction (SPE), liquid–liquid partitioning, column chromatography (e.g., silica gel, Sephadex, ion-exchange), preparative thin-layer chromatography (TLC), and high-performance liquid chromatography (HPLC) [83,84,85]. Advanced techniques like supercritical fluid extraction (SFE), counter-current chromatography (CCC), and flash chromatography have also gained popularity due to their efficiency and scalability [85,86]. The choice of method depends on the chemical properties of the target compound, such as polarity, molecular weight, and stability. Purification is often guided by bioassays, where active fractions are sequentially isolated and characterised to identify the bioactive principle [86].

## 3. Classical Approaches in NP Research

Traditional knowledge has long guided NP discovery, highlighting the therapeutic potential of various plant-derived bioactive compounds. This ethnobotanical wisdom laid the foundation for modern pharmacological research, inspiring scientists to isolate, characterise, and validate these compounds through systematic scientific inquiry [87]. The classical approach, a keystone of NP research, utilises bioactivity-guided fractionation and chromatographic separation to identify bioactive compounds [88]. Despite being time-consuming and labour-intensive, this method has been instrumental in discovering key drugs, including morphine (pain relief), paclitaxel (*Taxus brevifolia*, anticancer), and camptothecin (*Camptotheca acuminata*, anticancer) [89,90].

**Figure 1:**
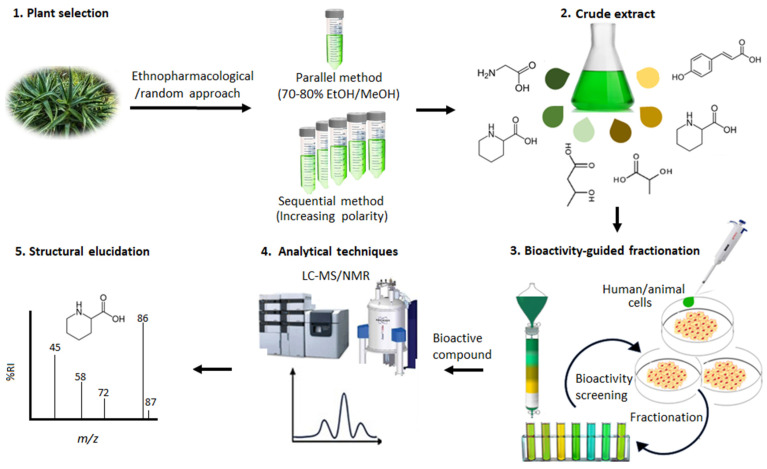
Illustration of the classical NP discovery approach. In this process, plant selection (**1**) is guided by random, ethnopharmacological, or ecological approaches. Extraction is performed using either sequential methods (involving solvents of varying polarities) or parallel methods (using 70–80% ethanol/methanol). The resulting crude extract (**2**) is fractionated and then subjected to cell-based biological screening assays (**3**) to isolate bioactive fractions. These fractions are further analysed using analytical techniques (**4**) s to identify and elucidate (**5**) the specific compounds responsible for the observed bioactivity.

As illustrated in Figure 1, the first step in NP discovery is selecting a plant source, which can follow three main strategies: (i) random selection, based on plant availability, (ii) ecological selection, guided by the plant’s ecological functions, and (iii) ethnopharmacological selection, where traditional medicinal use directs the choice [91,92]. Once selected, plants undergo extraction using solvents of varying polarities, such as ethanol or methanol for known bioactive compounds, or sequential solvent extraction (e.g., hexane → chloroform → methanol → water) when plant activity is unknown [93,94]. The resulting crude extracts are subjected to bioactivity-guided fractionation and screening, allowing the identification of potential therapeutic agents. The isolation process of NPs is guided by bioactivity screening, where the activity of isolated extracts is compared to crude extracts [6]. Any biological targets and sometimes mechanisms of action are evaluated through bioassays on animal or human cells and microorganisms, with primary screening assessing targets and efficacy and secondary screening focusing on mechanistic insights [95,96]. Active compounds undergo purification via column chromatography, followed by chemical characterisation using liquid chromatography–mass spectrometry (LC-MS) and nuclear magnetic resonance (NMR) [97,98]. Known compounds are identified by spectral matching, while unknowns are annotated through structural elucidation (Figure 1).

Over the years, traditional approaches for NP discovery have faced several significant barriers, including time-consuming processes, high rediscovery rates, and activity loss during fractionation [99]. The scarcity of medicinal plants and erosion of intergenerational ethnobotanical knowledge have further contributed to the declining pace of pharmacological advancements over the past 30 years. Classical approaches often lacked the efficiency, scalability, and precision needed for the rapid identification of novel bioactive compounds, resulting in gaps in chemical profiling and limited integration of complex biological data. To overcome these challenges, integrating ethnopharmacological knowledge with advanced omics technologies has the potential to transform NP discovery. Omics technologies are methods that analyse biological molecules on a large scale, providing comprehensive insights into an organism’s structure, function, and dynamics potential [78]. These technologies encompass a wide range of high-throughput analytical methods used to comprehensively study biological molecules and can prioritise promising plant candidates based on their inferred chemical profiles, enable rapid molecule identification, and incorporate genomic insights for more precise compound characterisation [100]. These innovations have streamlined high-confidence, automated NP identification, offering detailed compositional insights into medicinal plants, nutraceuticals, and botanicals with proven therapeutic potential [78,101].

## 4. Computational Metabolomics in Plant-Derived NP Research

**Figure 2:**
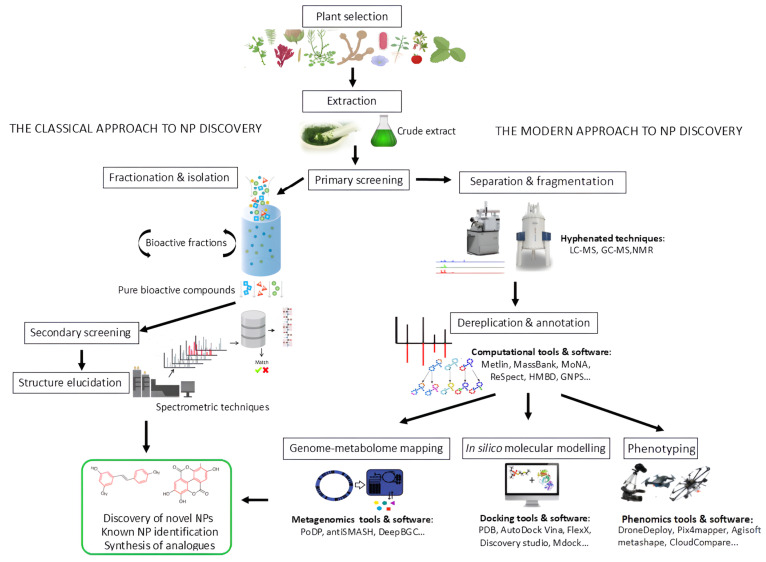
Schematic illustration of classical and modern approaches to natural product (NP) discovery. Both begin with plant selection through random, ecological, or ethnopharmacological approaches. Classical methods (left) rely on iterative fractionation and bioactivity screening followed by structure elucidation. Modern approaches (right) enable broader and deeper metabolome profiling, integrating advanced hyphenated technologies such as LC-MS and GC-MS, computational tools for dereplication and annotation (e.g., Metlin, MassBank, GNPS), genome–metabolome analysis (e.g., AntiSMASH, DeepBGC), in silico molecular docking (e.g., PDB, AutoDock Vina), and phenotyping (e.g., DroneDeploy), where drones equipped with specialised sensors and cameras capture high-throughput plant trait data, providing critical insights into plant growth, stress responses, and metabolite production.

The emergence of computational metabolomics has transformed plant-derived NP research, addressing key limitations of classical discovery methods. Computational metabolomics refers to the use of advanced data processing, machine learning, and bioinformatics tools to analyse complex metabolomic datasets [102,103]. This approach facilitates the identification, annotation, and functional interpretation of metabolites, overcoming the challenges of high data dimensionality and chemical diversity. By integrating advanced computational tools with omics sciences, researchers can now analyse complex plant metabolomes more comprehensively. Modern omics approaches now allow for much broader and deeper profiling of complex plant metabolome, providing richer datasets and more detailed insights into biochemical pathways [103,104]. The integration of advanced computational tools with metabolomics enables rapid annotation of metabolites and facilitates the prioritisation of bioactive compounds for further study. One notable advancement is the combination of metabolic profiling with bioactivity pattern analysis, which allows for the early-stage identification of functionally relevant molecules. This targeted strategy streamlines the process of NP isolation and characterisation, offering a more efficient and informed alternative to traditional methods (Figure 2) [105].

**Figure 3:**
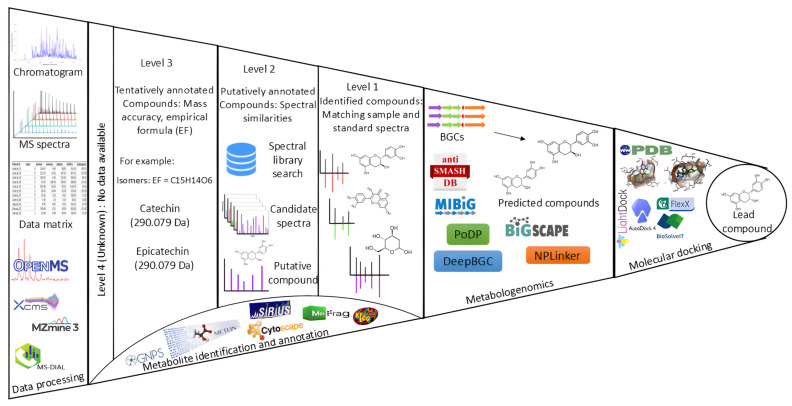
Computational metabolomics workflow for NP discovery. This figure illustrates the NP discovery pipeline (left to right), from data processing to lead compound identification. Chromatographic and MS data are analysed using tools like OpenMS, XCMS, and MZmine3, with metabolites classified into four levels: Level 4 (Unknown), Level 3 (Tentative annotation), Level 2 (Putative annotation), and Level 1 (Confirmed identification). Example compounds, catechin and epicatechin (C15H14O6, 290.079 Da), are used to demonstrate the process of distinguishing isomers. Identified metabolites are linked to biosynthetic gene clusters (BGCs) via tools like antiSMASH and MIBiG, which locate them in genomes and store validated clusters, respectively. Finally, molecular structures can undergo molecular docking (PDB, AutoDock, FlexX) to assess target interactions, supporting lead compound selection.

Metabolomics, together with the use of state-of-art technological equipment and artificial intelligence (AI)-inspired strategies, has inspired a renaissance of NP discovery [106,107]. This approach enables comprehensive qualitative and quantitative analysis of secondary metabolites using highly sensitive analytical techniques, such as (ultra) high performance liquid chromatography [(U)HPLC] and gas chromatography (GC) coupled to (high resolution) mass spectrometry [(HR)-MS] and NMR spectroscopy [108,109]. Among omics sciences, metabolomics is the most function-oriented, offering a direct biochemical snapshot of the plant’s metabolic state, shaped by both genetic and environmental factors [110,111]. The untargeted metabolomics workflow follows key steps—sample collection, data acquisition, annotation, and biological interpretation—all enhanced by computational tools for semi-automated NP identification (Figure 3).

### Technological Advancements in Metabolite Annotation

Technological advancements in metabolomics have transformed the study and discovery of plant-derived NPs, particularly by improving metabolite annotation and identification techniques. Metabolomics, which focuses on the comprehensive analysis of small molecules within biological systems, has progressed significantly through the integration of AI and machine learning (ML) across various stages of the workflow, from data acquisition to analysis [5,112]. Given the high dimensionality and complexity of metabolomics data, multivariate statistical analyses are widely used to extract meaningful biological insights. Techniques such as Principal Component Analysis (PCA) and Partial Least Squares Discriminant Analysis (PLS-DA) are used for data reduction, feature selection, and biomarker discovery [113,114]. When combined with computational metabolomics strategies, these statistical methods significantly improve data interpretation, bridging the gap between raw spectral data and biological insights.

Despite these advances, one of the main challenges in plant-based NP research has been the accurate identification of bioactive compounds due to the complexity and diversity of metabolites within biological samples. Only 2–10% of the spectra generated by MS in these studies are currently reliably annotated, leaving a vast amount of uncharacterised data known as “dark matter” [101,115]. Metabolite annotation itself is classified into multiple levels, as defined by the Metabolomics Standards Initiative (MSI) [116,117]. Level 1 refers to the identification of a metabolite with a reference standard, providing the highest confidence in annotation. Level 2 represents putative annotation, where the metabolite is identified based on spectral similarity to reference compounds but without direct confirmation by a reference standard. Level 3 involves the identification of compound classes, based on mass spectra and retention times, while Level 4 refers to unknown compounds with detectable mass features but without sufficient information for classification [118]. The majority of MS-based untargeted metabolomics annotations fall within Levels 2 to 4, underscoring the need for more robust tools to achieve higher annotation confidence.

Several advanced computational tools, listed in Table 3, have been developed to overcome the metabolite annotation challenges and improve annotation confidence, including moving more data from Levels 3 and 4 toward Levels 2 and 1 identification. The untargeted metabolomics pipeline begins with raw data processing and feature detection, which are critical for extracting meaningful mass spectral features. Tools like XCMS online [119], MZmine [120], OpenMS [121], and MS-DIAL [122] are widely used at this stage for peak detection, deconvolution, retention time alignment, and noise filtering. HomologueDiscoverer complements these tools by identifying and removing redundant or homologous features, streamlining datasets for downstream analysis [123]. Processed data are then explored using molecular networking techniques, which group related metabolites based on spectral similarity. The Global Natural Products Social Molecular Networking (GNPS) platform provides infrastructure for such analyses and supports workflows like Classical Molecular Networking (MN) and Feature-Based Molecular Networking (FBMN) [124,125]. While classical MN links only MS/MS spectral similarities, FBMN enhances resolution by incorporating MS1 feature alignment [124]. These networks are commonly visualised in Cytoscape (v 3.6), a powerful tool for mapping chemical relationships and functional annotations [126,127]. However, even with these advanced tools, the challenge of annotating unknown or novel compounds remains. Innovations like MS2LDA and its extension MS2LDA+ facilitate substructure-level annotation by identifying recurring fragmentation patterns (Mass2Motifs) [128,129]. This allows researchers to pinpoint chemical motifs across multiple samples, accelerating the discovery of abundant substructures in complex NP extracts [130]. In microbial NP research, MS2LDA was employed to discover both known and novel chemical motifs, facilitating the annotation of previously uncharacterised natural products [131]. This approach has been highly effective in NP research, such as in a study on *Fusarium oxysporum*, where FBMN enabled the discovery of novel isomers of beauvericin [124].

The GNPS molecular networking capabilities, combined with tools like MolNetEnhancer, have significantly improved the efficiency of metabolite annotation by linking spectral data to chemical ontologies [132]. MolNetEnhancer enriches molecular networks by incorporating chemical classification data from platforms like ClassyFire, allowing researchers to identify molecular families, subfamilies, and subtle structural variations within complex datasets. The GNPS platform can be further enhanced by additional tools like Spec2Vec and MS2Deepscore, which utilise unsupervised and supervised machine learning techniques to capture mass spectral similarities [133,134]. Spec2Vec, for instance, uses co-occurrence patterns in fragmentation data to identify related compounds, while MS2Deepscore uses supervised machine learning to train similarity scores based on the actual chemical similarities between molecules, improving the annotation of unknown metabolites [133,134]. These tools have expanded the scope of metabolomics, allowing researchers to explore previously uncharacterised NP chemical diversity more efficiently.

Network Annotation Propagation (NAP) further enhances molecular network annotations by propagating known metabolite information across networks, facilitating the identification of new or previously uncharacterised compounds [135]. DEREPLICATOR+ and VarQuest are also supportive in NP research, especially for microbial-derived compounds, as they enable the dereplication of known compounds by comparing mass spectra against extensive databases. DEREPLICATOR+, an enhanced version of DEREPLICATOR, improves the identification of known and novel natural products by incorporating additional structural insights [136]. VarQuest, in particular, has been used to identify novel antibiotic variants from *Streptomyces* species accelerating NP discovery by reducing redundancy in characterised metabolites [136]. MolDiscovery, another key tool from the same group, applies deep learning to predict MS/MS fragmentation spectra directly from molecular structures. It significantly improves annotation coverage and speed, especially for novel metabolites that lack reference spectra in existing databases [137]. SIRIUS + CSI provides another powerful tool for predicting molecular structures by combining high-resolution MS data with computational fragmentation models. This approach significantly enhances the accuracy of metabolite annotation, particularly for complex plant-derived NPs, by predicting fragmentation spectra in silico [132]. ClassyFire complements these tools by converting molecular descriptors, such as InChIKeys or SMILES, into hierarchical chemical categories. This chemical ontology classification has been invaluable for categorising newly discovered plant-based NPs and understanding their functional roles in plant metabolism [138].

Beyond structural annotation, prioritising metabolites with therapeutic relevance is essential. Tools such as Bioactivity-Based Molecular Networking (BBMN) [139], msFeaST [140], and FERMO [141] help integrate bioactivity data into the metabolomics workflow. BBMN links metabolite nodes in GNPS networks to experimental assay outcomes. msFeaST improves feature selection by combining MS1 and MS2 information across study conditions. FERMO ranks metabolite features based on their correlation with bioactivity profiles, supporting early-stage lead prioritisation. To fully harness omics data, tools like antiSMASH [142,143], plantiSMASH [144], and NPLinker [145] map annotated metabolites to biosynthetic gene clusters (BGCs), facilitating genome–metabolome integration. The Paired Omics Data Platform (PoDP) supports this effort by standardising links between omics datasets, streamlining NP discovery through metabologenomics [146].

In addition to GNPS, several databases have been indispensable in the metabolomics workflow. METLIN, the largest online repository of MS/MS data, enables researchers to match experimental spectra with reference data from known standards, aiding in the identification of metabolites from complex plant matrices [119]. MassBank and ReSpect also play pivotal roles in metabolite annotation. MassBank, with its spectral records from high-resolution MS platforms, is widely used for comparing experimental spectra, while ReSpect is specifically tailored to plant metabolomics, offering annotated spectra along with taxonomic information for the plants from which metabolites are derived [147]. The LOTUS database further enriches plant-based NP annotation by curating literature-based natural products and associating them with detailed taxonomic and chemical metadata, enabling the exploration of ethnobotanically relevant metabolites [148]. These databases have been valuable for NP research, particularly in traditional medicine, where many plant metabolites remain unexplored.

The integration of AI, ML, and advanced computational tools has had a transformative impact on NP research [149]. Studies using MN, ML algorithms, and spectral databases have led to the discovery of novel bioactive compounds in previously uncharacterised natural products, such as marine sponges and *Streptomyces* species, which produce metabolites with antimicrobial properties [136,150]. By leveraging the power of AI and ML, researchers have gained deeper insights into the chemical diversity of plant-based NPs and have significantly accelerated the process of NP discovery. These advancements not only streamline the metabolomics workflow but also pave the way for future breakthroughs in drug discovery, functional foods, and other industries reliant on natural products. At the same time, the integration of ethnobotanical knowledge into computational metabolomics presents an opportunity to further refine NP discovery. Many plant-derived NPs have origins in traditional medicine, and incorporating historical medicinal use data into computational pipelines can enhance the prioritisation of candidate metabolites. Existing tools such as MolNetEnhancer and ReSpect already classify metabolites based on taxonomic relationships, but extending their scope to include ethnobotanical metadata could offer valuable insights into NP functionality [132,147]. Additionally, genome–metabolome pairing (metabologenomics) can help link biosynthetic gene clusters (BGCs) to historically significant medicinal plants, refining the search for bioactive compounds with therapeutic potential [146,151]. Leveraging AI-driven text mining and ML algorithms to extract and integrate ethnobotanical data into NP research could facilitate the discovery of novel bioactives, bridging the gap between traditional knowledge and modern computational sciences.

**Table 3:** Summary of bioinformatic tools in the different steps of the computational metabolomics workflow for NP discovery, selected based on their widespread use and relevance in or promise for the field. The URL’s (website) in this table were accessed on the 13 November 2024.

Tool	Website	Description/Function/Role	References
**Data processing and analysis**			
XCMS online	https://xcmsonline.scripps.edu	Nonlinear retention time alignment	[119]
MZmine	https://github.com/mzmine/mzmine	Mass detection, peak deconvolution, retention time alignment	[120]
OpenMS	https://www.openms.de/	Peak picking, retention time alignment, baseline and noise filtering, metabolite quantification and identification	[121]
MS-DIAL	https://systemsomicslab.github.io/compms/msdial/main.html	Spectral deconvolution, peak identification, statistical analysis	[122]
HomologueDiscoverer	https://github.com/kevinmildau/homologueDiscoverer	Detection and omission of noise features and redundant features	[123]
**Metabolite annotation libraries**			
METLIN	https://metlin.scripps.edu/	Repository for searchable MS2 data (positive and negative modes) and neutral loss libraries acquired from standards	[152]
MassBank	https://massbank.eu/MassBank/	Spectral data repository	[153,154]
ReSpect	https://github.com/shahab-sarmashghi/RESPECT	Spectra and taxonomy information repository	[147]
GNPS	https://gnps.ucsd.edu/	Repository for spectral libraries, molecular network construction	[125]
**Mass Spectral Networking, Embedding and Annotation**			
Classical Molecular Networking (MN)	https://gnps.ucsd.edu/	Groups metabolites based on MS/MS spectra similarity, forming molecular networks	[125]
Feature-Based Molecular Networking (FBMN)	https://gnps.ucsd.edu/	Enhances MN by using MS1 feature data to align nodes more precisely in molecular networks	[124]
msFeaST	https://github.com/kevinmildau/msFeaST	Integrates MS1 and MS2 information for improved feature selection in molecular networking	[140]
MS2LDA	https://ms2lda.org/	Decomposition of molecular fragmentation, annotation and discovery of Mass2Motifs	[128]
MS2Query	https://github.com/iomega/ms2query	Integrates Spec2Vec and MS2Deepscore, ranks potential analogues and exact matches	[155]
Spec2Vec	https://github.com/iomega/spec2vec	Assess and rank spectral similarities	[133]
MS2Deepscore	https://github.com/matchms/ms2deepscore	Predicts structural similarity between MS/MS spectra	[134]
NAP	https://gnps.ucsd.edu/ProteoSAFe/static/gnps-splash.jsp	Improves in silico fragmentation candidate structure ranking	[135]
DEREPLICATOR	https://gnps.ucsd.edu/	In silico identification of both peptidic and non-peptidic natural products	[136]
SIRIUS+CSI:FingerID	https://github.com/computational-metabolomics/sirius-csifingerid-galaxy	Analysis of isotope patterns, compound class prediction.	[156,157]
ClassyFire	https://bio.tools/ClassyFire	Large-scale automated chemical/metabolite classification	[138]
MolNetEnhancer	https://gnps.ucsd.edu/	Enhances molecular networks by integrating chemical classification data to assign chemical ontologies to metabolites. Helps identify molecular families and structural variations	[132]
Bioactivity-Based Molecular Networking (BBMN)	https://gnps.ucsd.edu	Links molecular networks to bioactivity data to discover bioactive compounds	[139]
FERMO	https://fermo.bioinformatics.nl	Links bioactivity information to metabolite features in natural product discovery	[141]
Cytoscape	https://cytoscape.org/	Network data integration, analysis, and visualisation	[127]
**Metabologenomics**			
antiSMASH	https://github.com/antismash/antismash	Identify, annotate, and compare gene clusters that encode the biosynthesis of NPs	[142]
Paired Omics Data Platform (PoDP)	https://pairedomicsdata.bioinformatics.nl	Standardise links between genomic and metabolomics data in a computer readable format to further the field of natural products discovery	[146]
plantiSMASH	http://plantismash.secondarymetabolites.org/	A specialised version of antiSMASH designed for the identification, annotation, and analysis of BGCs in plant genomes	[144]
NPLinker	https://github.com/nplinker/nplinker	Links BGCs with metabolomics data to facilitate the discovery of natural products by integrating and analysing paired omics datasets	[158]

## 5. Integration of Omics Technologies for NP Discovery

In the quest to bridge traditional knowledge with modern scientific innovation, the integration of omics technologies has emerged as a transformative approach in the discovery of plant-derived NPs [159,160]. Fuelled by the technological advances in drug discovery research (i.e., computational metabolomics), the global demand for herbal medicines continues to rise steadily each year. However, large-scale production of medicinal plants and their derivatives remains limited [161,162]. By integrating ethnobotanical knowledge with omics data, researchers can prioritise plants with historical medicinal relevance, thereby increasing the likelihood of identifying novel bioactive compounds. Recent advances in paired-omics strategies have further enhanced our ability to link genes, transcripts, and metabolites, providing multiple layers of evidence for biosynthetic pathway discovery. Unlike single-omics approaches, paired-omics integrates multiple data types within the same experimental framework, allowing researchers to map biosynthetic pathways with greater precision [163,164]. This approach is particularly useful in plants, where genome complexity and chemical diversity present challenges in pathway elucidation. Experimental designs incorporating paired-omics methods are increasingly being optimised to enhance NP discovery and functional annotation [165]. Such integrative multi-omics frameworks have the potential to revolutionise biopharmaceutical sciences.

### 5.1. Genomics: Uncovering Biosynthetic Potential

Genomics serves as the cornerstone of NP discovery by offering a comprehensive map of an organism’s genetic potential. By identifying and analysing BGCs, researchers can predict the repertoire of secondary metabolites that an organism can produce [166]. In the realm of plant-derived NPs, genomics has played a pivotal role in uncovering the genes responsible for the biosynthesis of complex molecules such as alkaloids, terpenoids, and flavonoids [167,168]. The advent of advanced sequencing technologies, particularly next-generation sequencing (NGS), has dramatically accelerated the discovery of BGCs, enabling researchers to delve deeper into the genetic diversity within and across plant species [169,170].

Genomics not only facilitates the discovery of new compounds but also sheds light on the evolutionary processes that have shaped the rich diversity of secondary metabolites in plants. In a study conducted by Johnston et al., bacterial genome sequences were meticulously analysed to identify BGCs encoding the enzymatic machinery necessary for NP biosynthesis [171]. This genomics-driven approach led to the discovery of several novel NPs, including acidobactins, vacidobactins, variobactins, and potensimicin, from bacterial strains *Acidovorax citrulli*, *Variovorax paradoxus*, and *Nocardioides potens* [172]. These newly identified compounds exhibit promising medicinal properties, including potential antimicrobial and anticancer activities, highlighting the power of genomics in uncovering bioactive molecules with therapeutic potential.

### 5.2. Transcriptomics: Understanding Gene Expression Dynamics

While genomics offers a static blueprint of an organism’s biosynthetic potential, transcriptomics provides a dynamic view by analysing gene expression patterns across different conditions [173]. This approach allows researchers to identify which genes within BGCs are actively transcribed and how their expression is regulated in response to environmental stimuli, developmental stages, or stress conditions [173,174]. Understanding these regulatory networks is crucial for elucidating the mechanisms that govern NP biosynthesis. RNA sequencing (RNA-Seq) is a powerful transcriptomics tool that quantifies gene expression levels across the entire genome, offering insights into the temporal and spatial dynamics of NP production [169,173]. Transcriptomic data can reveal the activation of specific biosynthetic pathways in response to particular stimuli, thereby guiding the experimental conditions necessary to induce the production of target NPs.

A practical application of transcriptomics in NP discovery is exemplified by MEANtools, a computational workflow that systematically integrates transcriptomics and metabolomics to predict candidate metabolic pathways [175]. This tool effectively reconstructed the falcarindiol biosynthetic pathway in tomatoes, demonstrating how transcriptomic data can be leveraged to map metabolite–transcript correlations and predict enzymatic reactions [170,175]. Additionally, the potential of transcriptomics in NP discovery is exemplified by the study where, using RNA-Seq, the researchers analysed the transcriptomes of several Chinese medicinal plants, focusing on identifying genes involved in the biosynthesis of key secondary metabolites [52]. Their study led to the identification of differentially expressed genes linked to the production of bioactive compounds, including flavonoids, alkaloids, and terpenoids. The study uncovered the upregulation of genes associated with the biosynthesis of berberine, a well-known alkaloid with antimicrobial properties, in *Coptis chinensis* (Huanglian) [52]. These findings align with recent integrative omics approaches, which have further demonstrated the effectiveness of transcriptomics in NP pathway elucidation, particularly through unsupervised network-based correlation methods that capture the interplay between gene expression and metabolite production [170,175].

### 5.3. Proteomics: Linking Genotype to Phenotype

Proteomics serves as a critical bridge between transcriptomics and metabolomics by providing direct evidence of protein expression, modification, and function. In the context of NP discovery, proteomics is essential for connecting genetic and transcriptional information to the actual biosynthetic processes occurring within an organism [176]. Mass spectrometry (MS)-based proteomics is at the forefront of this approach, enabling the identification and quantification of proteins, particularly enzymes, that are involved in NP biosynthesis [177,178]. By analysing the proteome, researchers can confirm which enzymes are present and active in the biosynthetic pathways, offering valuable insights into the functional roles these proteins play in producing specific NPs. Furthermore, proteomics can reveal post-translational modifications that may regulate enzyme activity, adding another layer of complexity and regulation to NP biosynthesis [179]. This comprehensive approach is particularly useful for characterising the entire biosynthetic pathway of a NP, from gene expression to protein activity and ultimately to metabolite production.

A compelling example of this approach is the discovery of koranimine, a novel cyclic imine natural product, through proteomics-based research. In this study, proteomics was employed to investigate the protein expression profiles of the marine sponge *Theonella swinhoei*, the source material from which koranimine was isolated [180]. Mass spectrometry played a crucial role in identifying the biosynthetic enzymes responsible for koranimine production, thereby effectively linking the genetic information to the actual production of this NP. This example underscores the power of proteomics in unravelling the complex biosynthetic pathways of natural products, offering a clear connection from genotype to phenotype.

While single-omics approaches such as genomics, transcriptomics, and proteomics have individually provided significant insights into NP discovery, they each have inherent limitations when applied independently. Genomics, while powerful in identifying BGCs and predicting biosynthetic potential, offers a static view of an organism’s genetic capacity without revealing which genes are actively involved in NP production under specific conditions [181]. Transcriptomics, on the other hand, provides dynamic data on gene expression but does not confirm whether the proteins necessary for NP biosynthesis are produced or functional [52,173]. Similarly, proteomics, although invaluable for identifying active enzymes and post-translational modifications, does not provide information about the upstream regulatory mechanisms that govern gene expression or the downstream metabolic products [168,178].

### 5.4. Applications of Integrated Omics in NP Discovery

**Figure 4:**
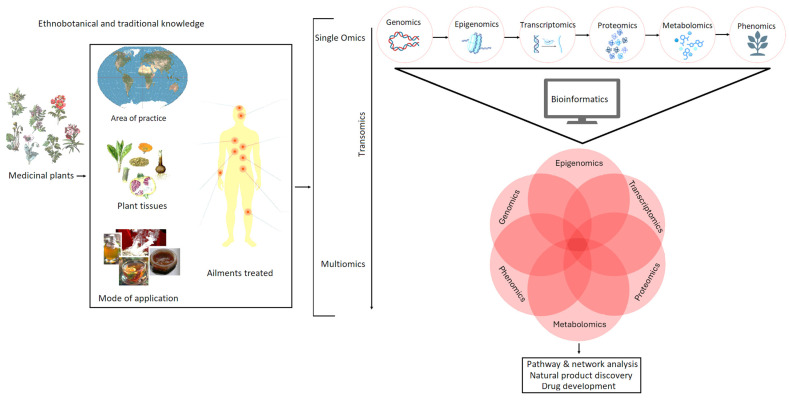
Ethnobotanical knowledge and multi-omics integration in NP discovery. This figure illustrates how ethnobotanical and traditional knowledge informs omics sciences for NP discovery. Insights from traditional medicinal plants, area of practice, plant tissues used, their mode of application and ailments they treat, guide single-omics studies (genomics to phenomics), which are integrated through bioinformatics into a multi-omics framework. This enables pathway and network analysis, facilitating the discovery of bioactive compounds and accelerating drug development.

Integrated omics—also referred to as multi-omics—has emerged as a transformative strategy in NP discovery (Figure 4), enabling a comprehensive, systems-level understanding of biological processes underpinning specialised metabolism. Since its emergence in the early 2000s, the paradigm has expanded from isolated single-omics approaches to integrated analyses that unify genomic, transcriptomic, proteomic, and metabolomic layers [162,182]. By bridging the gap between genetic potential and chemical output, multi-omics facilitates the efficient identification and characterisation of novel bioactive compounds across diverse biological systems, including plants, microbes, and marine organisms [14,183]. One of the earliest successful applications of integrated omics in NP discovery was demonstrated by Sun et al. (2013) [183], who used RNA-Seq to investigate the transcriptomic responses of Taxus cells exposed to elicitors. This enabled the identification of key genes involved in paclitaxel biosynthesis and their regulation under different stimuli. Similarly, proteogenomic approaches—such as those used by Bumpus et al. (2009)—have proven powerful in microbial NP discovery [179]. By integrating mass spectrometry-based proteomics with genomic mapping, they linked expressed biosynthetic enzymes (e.g., NRPS and PKS) to their biosynthetic gene clusters (BGCs), revealing previously undetected metabolic pathways.

The integration of metabolomics and genomics, or metabologenomics, has further enhanced NP discovery. This approach leverages co-occurrence-based correlations and molecular networking to associate metabolite MS/MS features with their cognate BGCs [184]. Tools like NPLinker, when combined with genome mining platforms such as antiSMASH and plantiSMASH, have demonstrated high accuracy in linking genotypes to phenotypes [142,145,158]. In marine cyanobacteria, Leão et al. (2021) combined genomics and LC-MS/MS metabolomics to identify lipid-like metabolites linked to promising BGCs across 24 strains [185]. Despite these advances, metabologenomics in plants remains relatively underexplored. This is primarily due to the structural complexity and dispersed nature of plant BGCs. However, recent paired-omics strategies integrating transcriptomics and metabolomics have improved pathway discovery, particularly in medicinal plants with challenging genomes [165]. These approaches are increasingly supported by curated repositories like the Paired Omics Data Platform (PoDP), which facilitates the integration of standardised multi-omics datasets [146].

Crucially, incorporating ethnobotanical knowledge into these frameworks has emerged as a powerful addition to NP discovery (Figure 4). Ethnobotanical data—encompassing traditional plant uses, cultural practices, and indigenous taxonomies—can rationally guide species and tissue selection, focusing omics efforts on candidates with known therapeutic applications. This knowledge-driven sampling enhances the efficiency of NP discovery by narrowing down chemical search space using historical human–plant interaction data [186]. In the study of Qiao et al., researchers applied transcriptomic and metabolomic profiling to medicinal Scutellaria species used in traditional medicine [186]. Their work uncovered lineage-specific flavone biosynthetic pathways, revealing enzymes involved in structural diversification and pharmacological enhancement.

Bioinformatics tools like FERMO enable prioritisation of metabolite features by integrating molecular data with bioactivity assays, taxonomy, and ethnobotanical context [141]. This allows researchers to triage metabolite candidates not only by abundance or statistical relevance but also by cultural or historical significance. Ontologies like ClassyFire and resources like the LOTUS database complement these efforts by providing ontological classifications and taxonomic information that enrich metabolite annotations with contextual information [138,148]. Multi-omics databases further support discovery efforts, for example, the Ginseng Genome Database offers a complete spectrum of genomic, transcriptomic, proteomic, and metabolomic data for *Panax ginseng* [187]. Other repositories, such as HMOD, GPGP, MPOD, and BPGD, collectively catalogue omics information for hundreds of medicinal plants [188,189,190,191].

Despite these successes, integrating and interpreting multi-omics data presents significant challenges. These include high dimensionality, data heterogeneity, inconsistent measurement methods, and a lack of standardised formats [192,193]. Biological complexity and dynamic system states introduce variability that complicates data modelling and integration. Additionally, interpretation often requires domain expertise and experimental validation, particularly in pharmacognosy and drug discovery [194,195,196]. Addressing these limitations necessitates rigorous data management protocols, transparent reporting, and the adoption of best practices. Sophisticated computational frameworks must be developed to reduce bias, handle uncertainty, and extract meaningful insights. Only through such systemic improvements can integrated omics realise its full potential in accelerating the discovery, validation, and development of natural products for human health.

## 6. Conclusions and Future Perspectives

The integration of ethnobotanical knowledge with multi-omics approaches represents a paradigm shift in NP discovery, bridging centuries of traditional medicine with state-of-the-art scientific methodologies. Traditional healing systems, including Ayurveda, TCM, and Indigenous medicine, have long provided valuable insights into medicinal plant use; yet the lack of rigorous standardisation, validation, and mechanistic understanding has limited their full clinical potential. By leveraging multi-omics methods, modern NP research can start to systematically decode plant biosynthetic pathways and elucidate the molecular underpinnings of their bioactivity. While bioactivity-guided fractionation has historically been instrumental in NP identification, it is inherently labour-intensive, low-throughput, and prone to rediscovering known compounds. The advent of computational omics, AI-driven analytics, and ML models has dramatically enhanced the speed, accuracy, and depth of metabolite annotation, enabling high-throughput screening and precision-driven compound identification. Furthermore, metabologenomics, which integrates BGCs with metabolomic data, provides a more comprehensive, predictive framework for discovering novel bioactive compounds and their biosynthetic pathways.

Despite these advances, key challenges persist, including data heterogeneity, computational scalability, and the integration of complex biological datasets. Addressing these issues necessitates the development of robust analytical pipelines, standardised data curation practices, and interdisciplinary collaboration between computational scientists, biochemists, and ethnopharmacologists. Moving forward, future research should prioritise the refinement of bioinformatics tools for multi-omics data integration, experimental validation of computational predictions, and the bioprospecting of underexplored medicinal plants. Additionally, establishing a scientifically rigorous yet culturally inclusive framework for validating traditional remedies is critical to harmonising traditional and modern medicine. We anticipate that by merging ethnobotanical wisdom with advanced omics technologies, NP research can unlock unprecedented opportunities for drug discovery, sustainable bioprospecting, and the development of next-generation therapeutics.

## Data Availability

No new data were created or analyzed in this study.
